# Aflatoxins in Food and Feed: An Overview on Prevalence, Detection and Control Strategies

**DOI:** 10.3389/fmicb.2019.02266

**Published:** 2019-10-04

**Authors:** Dipendra K. Mahato, Kyung Eun Lee, Madhu Kamle, Sheetal Devi, Krishna N. Dewangan, Pradeep Kumar, Sang G. Kang

**Affiliations:** ^1^School of Exercise and Nutrition Sciences, Deakin University, Burwood, VIC, Australia; ^2^Molecular Genetics Laboratory, Department of Biotechnology, Yeungnam University, Gyeongsan, South Korea; ^3^Department of Forestry, North Eastern Regional Institute of Science and Technology, Nirjuli, India; ^4^SAB Miller India Ltd., Sonipat, India; ^5^Department of Agricultural Engineering, North Eastern Regional Institute of Science and Technology, Nirjuli, India

**Keywords:** human health, outbreaks, aflatoxins contamination, detection, food and feed

## Abstract

Aflatoxins produced by the *Aspergillus* species are highly toxic, carcinogenic, and cause severe contamination to food sources, leading to serious health consequences. Contaminations by aflatoxins have been reported in food and feed, such as groundnuts, millet, sesame seeds, maize, wheat, rice, fig, spices and cocoa due to fungal infection during pre- and post-harvest conditions. Besides these food products, commercial products like peanut butter, cooking oil and cosmetics have also been reported to be contaminated by aflatoxins. Even a low concentration of aflatoxins is hazardous for human and livestock. The identification and quantification of aflatoxins in food and feed is a major challenge to guarantee food safety. Therefore, developing feasible, sensitive and robust analytical methods is paramount for the identification and quantification of aflatoxins present in low concentrations in food and feed. There are various chromatographic and sensor-based methods used for the detection of aflatoxins. The current review provides insight into the sources of contamination, occurrence, detection techniques, and masked mycotoxin, in addition to management strategies of aflatoxins to ensure food safety and security.

## Introduction

Food safety and security are among the major problems in the current climate of increasing population. These are mainly determined by three key aspects viz., (i) enough food availability, (ii) access to safe food and (iii) utilization of the food in terms of quality, nutritional and cultural purposes for a healthy life (FAO, 1996). The failure of any of these aspects leads to food insecurity and malnutrition that further influences human health, in addition to the socio-economic aspect of society. In addition, food and feed contamination by mycotoxins are one of the key factors responsible for creating food insecurity (Udomkun et al., 2017).

As per the Food and Agriculture Organization (FAO), one-fourth of the world’s crop is affected by mycotoxins (Wu, 2007; Pankaj et al., 2018). The three main genera of fungi producing mycotoxins are *Aspergillus*, *Fusarium*, and *Penicillium* (Reddy et al., 2010). Among various type of mycotoxins, aflatoxins (AFs) are highly toxic and are known to contaminate a wide variety of foods such as maize, groundnuts, dried fruits, meat and milk-based products (Mutegi et al., 2009; Perrone et al., 2014; Iqbal et al., 2015). AFs are produced by *Aspergillus* species, namely *A. flavus*, *A. nomius* and *A. parasiticus* (Payne and Brown, 1998), in addition to its production by other species of *Aspergillus* like *A. astellatus* (Reiter et al., 2009). These fungi usually grow in the warm and humid conditions of tropical and subtropical regions (Magan and Aldred, 2007; Battilani et al., 2011). Food processing techniques are not sufficient to eliminate AFs from contaminated food and feed due to their heat resistant nature (Medina et al., 2017b).

The ingestion of AFs from contaminated food and feed has led to serious health complications in humans and animals (Fung and Clark, 2004; Binder et al., 2007; Sherif et al., 2009). Therefore, different countries have implemented strict regulations for AFs in food and feed to maintain the health of individuals (Juan et al., 2012). The safe limit of AFs lies in the range of 4–30 μg/kg for human consumption. The European Union has the strictest standard level with AFB1 and total AFs not beyond 2 μg/kg and 4 μg/kg, respectively, in any product meant for direct consumption (EC, 2007, 2010). Similarly, the maximum acceptable limit set for AFs in the United States is 20 μg/kg (Wu, 2006). Besides this, various innovative technologies and control strategies are applied for pre- and post-harvest management of AFs to enhance sustainable agricultural productivity (Prietto et al., 2015). Though there are numerous publications on AFs in food and feed, the novelty and strength of this review lie with the enlistment of the new methods developed for AFs detection in food and feed with special reference to masked AFs. In addition, the review also focuses on the occurrence, impact of climate change along with the control strategies of AFs in food and feed to ensure food safety and security for healthy living and socio-economic development.

## Occurrence of Aflatoxins in Food and Feed

Aflatoxins are chemically difuranocoumarin derivatives with a bifuran group attached to the coumarin nucleus and a pentanone ring (in case of AFBs) or a lactone ring (in case of AFGs) (Schuda, 1980). The four major AFs among the identified 20 are AFB1, AFB2, AFG1 and AFG2. The B-types are produced by *A. flavus* while G-types are produced by *A. parasiticus* (Kumar et al., 2017). The biosynthesis of AFs consists of 18 enzymatic steps with at least 25 genes responsible for producing the enzymes and regulating the biosynthetic process (Yu et al., 2002; Yabe and Nakajima, 2004).

The occurrence of AFs is common in wide varieties of food and feed (Table 1). Some of the most affected food and feed include peanuts, nuts, figs, corn, rice, spices and dried fruits (Martinez-Miranda et al., 2019). It has been shown that among the tested cereals, 37.6% were at least contaminated by any of the AFs (Andrade and Caldas, 2015). Though rice is not the high-risk commodity for AFs contamination, but AFB1 besides other mycotoxins have been detected in rice from China, Egypt, India, Iran, Malaysia, Nepal, Pakistan, Philippines, United Kingdom and United States (Tanaka et al., 2007; Rahmani et al., 2011; Lutfullah and Hussain, 2012). Therefore, AFs pose serious health issues by their ingestion from contaminated food and feed or by carryover AFs in them (Nordkvist et al., 2009; Reiter et al., 2010).

**TABLE 1:** Occurrence of Aflatoxins in food and feed around the world.

**Country**	**Food matrix**	**Aflatoxin**	**Range (μg/kg)**	**Detection technique**	**References**
Turkey	Almond	AFB1	1–13	TLC	Gürses, 2006
Turkey	Butter	AFM1	<0.001–0.100	ELISA	Aycicek et al., 2005
Brazil	Cashew nuts	Total AFs	0.60–31.50	ELISA	Milhome et al., 2014
United States	Chilies	AFB1	<2	ELISA and TLC	Singh and Cotty, 2017
Costa Rica	Corn	Total AFs	24	ELISA and HPLC	Granados-Chinchilla et al., 2017
Zimbabwe	Corn	AFB1	0.75–26.6	HPLC	Murashiki et al., 2017
India	Corn	AFB1	48–383	HPLC	Mudili et al., 2014
Serbia	Corn	Total AFs	1.01–86.10	ELISA	Kos et al., 2013
Vietnam	Corn	AFB1	1.0–34.80	ELISA	Lee et al., 2017
Turkey	Cream cheese	AFM1	0.1–0.70	ELISA	Yaroglu et al., 2005
Pakistan	Dried Fruits	AFB1	0.04–9.80	HPLC	Masood et al., 2015
Turkey	Feed	AFB1	0–5	LCMS/MS	Yalcin et al., 2017
Turkey	Figs	Total AFs	0.1–28.20	HPLC	Kabak, 2016
Nigeria	Ginger	Total AFs	0.11–9.52	HPLC	Lippolis et al., 2017
Ethiopia	Groundnuts	Total AFs	15–11,900	HPLC	Chala et al., 2013
Turkey	Hazelnut	AFB1	0.07–43.60	HPLC	Baltaci et al., 2012
Serbia	Infant formula	AFM1	<0.03–0.02	HPLC	Torović, 2015
Turkey	Lentil	AFB1	0.57–1.74	HPLC	Baydan et al., 2016
Turkey	Maize flour	AFB1	0.041–1.12	HPLC	Kara et al., 2015
Egypt	Meat products	Total AFs	0.47–2.10	Fluorimeter	Abd-Elghany and Sallam, 2015
Greece	Milk	AFM1	<0.005–0.02	ELISA	Tsakiris et al., 2013
Iran	Milk (cow)	AFM1	0.006–0.18	HPLC	Bahrami et al., 2016
Brazil	Milk (cow)	AFM1	0.05	HPLC	Picinin et al., 2013
Italy	Milk (cow/buffalo)	AFM1	0.004	HPLC	De Roma et al., 2017
Portugal	Milk (cow)	AFM1	0.005–0.07	ELISA	Duarte et al., 2013
Japan	Nuts	AFB1	0.17–2.59	HPLC, HPTLC	Kumagai et al., 2008
Saudi Arabia	Nuts	Total AFs	1.0–110	HPLC	Neamatallah and Serdar, 2013
Malawi	Nut-based foods	AFB1	0.1–40.60	HPLC	Matumba et al., 2014
Zambia	Peanuts	AB1	0.015–46.60	HPLC	Bumbangi et al., 2016
Taiwan	Peanut products	Total AFs	0.2–513.40	HPLC	Chen et al., 2013
Turkey	Red-chili powder	AFB1	0.025–40.90	ELISA	Aydin et al., 2007
China	Rice	AFB1	0.03–20	HPLC	Lai et al., 2015
India	Rice	AFB1	0.1–308	Indirect competitive (icELISA)	Reddy et al., 2009
Pakistan	Rice	AFB1	0.04–21.30	HPLC	Iqbal et al., 2016
China	Rice	AFB1	0.1–136.80	HPLC	Sun et al., 2011
Tunisia	Sorghum	AFB1	0.4–25.1	HPLC	Ghali et al., 2010
Italy	Spices	AFB1	0.59–5.38	HPLC	Prelle et al., 2014
Malaysia	Spices	AFB1	0.58–4.64	ELISA	Reddy et al., 2011
Tunisia	Wheat	AFB1	0.12–18	HPLC	Ghali et al., 2010
Malaysia	Wheat	AFB1	0.55–5.07	ELISA	Reddy et al., 2011
China	Yogurt	AFM1	0.05	HPLC	Guo et al., 2013
Iran	Yogurt	AFM1	0.006–0.021	HPLC	Bahrami et al., 2016

AFB1, as a potent carcinogen to humans, is associated with serious health complications (IARC, 2012). It has been a causal factor for liver cancer and acute hepatitis as well as periodic outbreaks of acute aflatoxicosis leading to death (Azziz-Baumgartner et al., 2005) as reported with lethal aflatoxicosis in Kenya (Probst et al., 2007). AFs are mostly detoxified in the liver which is the reason why liver cancer is rare. After the ingestion of AFB1, a series of metabolic processes converts it to an active intermediate, AFB1-exo-8,9-epoxide, by cytochrome P450 enzymes. The detoxification reaction occurs in conjugation with glutathione transferases (GSTs). The detoxification mechanism of AFB1-exo-8,9-epoxide might be linked to the mechanism that prevents liver cancer, however, it is not yet fully revealed (Guengerich et al., 1998). Unfortunately, on the other hand, the food and feed contamination by AFs is a persistent problem worldwide. The outbreaks due to AFs are more prone in tropical and subtropical areas, with a few in temperate regions (like the United States Midwest). In addition, the Mediterranean zones have become prone to AFs contamination due to shifting in traditional occurrence areas of AFs because of climate change i.e., increase in average temperatures, CO_2_ levels and rainfall patterns (Marasas et al., 2008). This has led to an increase in contamination of crops with fungi and AFs worldwide.

## Crops Affected by Aflatoxins

Cereals and cereal-based products are the major foods for human consumption worldwide (Temba et al., 2017). Among cereals, rice and corn are mostly contaminated by AFs in natural conditions due to changes in agricultural practices. The AFs are produced both in pre- and post-harvest conditions (Hesseltine, 1974). Filazi and Sireli (2013) reported rice to be more prone to AFs contamination as compared to other cereals. The fungal growth occurs due to improper drying of rice grains retaining higher moisture content (>14%). As a result, these fungi cause discoloration of grain and/or husk along with deteriorating the quality of the grains. Groundnut and beans, on the other hand, are frequently used in many African diets to supplement cereal diets (Soro-Yao et al., 2014). However, these are highly prone to AFs contamination both in field and storage conditions (Lombard, 2014). The extent of fungal growth and AFs production in cereals depends on temperature, moisture, soil type, and storage conditions (Achaglinkame et al., 2017). In addition, spices are susceptible to AFs contamination and are significantly affected by storage and processing conditions. Elshafie et al. (2002) reported the AFs contamination in a wide variety of spices including black pepper, cardamom, cinnamon, clove, cumin, coriander, and ginger in the Sultanate of Oman. Furthermore, Tchana et al. (2010) reported the presence of AFs in eggs collected from a poultry farm and in raw cow milk in Cameroon. Hence, the affected crops allow AFs to enter the food chain, which is very much influenced by the climatic conditions.

## Impact of Climate Change on Aflatoxin Production

Climate change significantly impacts on the quality and availability of staple foods for consumption. With the increasing population worldwide, a major emphasis has been put on the safety of food and feed that can address the increasing demand with the increase in the yields by protecting the crops from adverse climatic conditions (Medina et al., 2017a). Aflatoxins contamination has affected millions of hectares of maize and peanut crops in the United States (Robens and Cardwell, 2003). Maize is a staple food for people living in warm climates throughout Asia, Africa, and the Americas, which are prone to the influences of climate change (Lewis et al., 2005). The change in climate simultaneously impacts the complex communities of AF-producing fungi by altering the number of AF-producers to change its fungal community’s structure. Aflatoxins contamination occurs via an initial phase during crop development and a second phase during crop maturation. The contamination is greater in warm, humid, and even hot deserts and drought conditions (Cotty and Jaime-Garcia, 2007).

*A. flavus* has highly evolved physiological mechanisms to acclimatize to adverse climatic conditions and dominates other fungal species (Nesci et al., 2004; Magan, 2007). Climate change alters the temperature and water activity (a_w_) in the environment which further influences the gene expression to produce AFs. The conditions of temperature and a_w_ regulate the extent of fungal growth and AFs production (Schmidt-Heydt et al., 2009; Schmidt-Heydt et al., 2010). The AF-producing genes are grouped on the genome and express the main regulatory genes (aflR; aflS), as well as structural genes (aflD) which are influenced by the interaction of temperature × a_w_ conditions. As revealed by Schmidt-Heydt et al. (2010), the expression proportion of aflR/aflS significantly correlates with the amount of AFB1 produced. In addition, the expression of sugar transporter genes was significantly affected by the condition of temperature and a_w_ (Medina et al., 2014; Medina et al., 2015). Further, Bernáldez et al. (2017) studied the effect of interactions of temperature and a_w_ on the biosynthetic regulatory gene (aflR) expression and production of AFB1 by *A. flavus* in maize. They observed the optimum growth of *A. flavus* at 30°C/0.99 a_w_ with no growth at 20°C/0.90 a_w_. Both temperature and a_w_ influenced the relative aflR gene expression and AFB1 production, however, the trends for the production of AFB1 were not in accordance with the gene expression. Further, the effect of temperature (20, 27, and 35°C) and a_w_ (0.82, 0.86, 0.90, 0.94, and 0.98) on the growth of *A. flavus* and *A. parasiticus* along with the production of AFB1 were investigated on ground Nyjer seeds by Gizachew et al. (2019). The maximum AFB1 production was observed at 27°C/0.90 a_w_ for both *A. flavus* and *A. parasiticus*. In addition to this, the fungi showed optimum growth on polished rice in the range of 28–37°C/0.92–0.96 a_w_. The maximum AFB1 was produced at 33°C/0.96 a_w_ (Lv et al., 2019). Based on the investigation by Battilani et al. (2016) on the possible emergence of AFB1 in cereals in the European Union as a result of climate change, for every 2°C increase in temperature, there is an increase in AFs risk in the various regions of Spain, Italy, Greece, Portugal, Bulgaria, Albania, Cyprus and Turkey. The risk for AFs contamination in maize is likely to increase in Europe due to favorable climatic conditions for *A. flavus* in the next 30 years (Moretti et al., 2019). Therefore, proper detection methods and control strategies are crucial to combat the burning issues of AFs in food and feed.

## Detection Methods

The detection of AFs has been performed by the Association of Official Analytical Chemists (AOAC) official method in food and feed samples (Kumar et al., 2017). Among the most commonly employed methods are chromatographic methods like thin layer chromatography (TLC) (Fallah et al., 2011), high performance liquid chromatography (HPLC) and liquid chromatography mass spectroscopy (LCMS) (McDanell et al., 1988; Samarajeewa et al., 1991; Herzallah, 2009), besides the enzyme-linked immunosorbent assay (ELISA) (Tabari et al., 2011; Andrade et al., 2013; Sulyok et al., 2015). However, the drawbacks of these standard methods are that they are unsuitable for rapid and real-time applications in food and feed samples as they are tedious, time-consuming and require skilled personnel to operate. Therefore, rapid and robust methods like polymerase chain reaction (PCR) and non-destructive methods based on fluorescence/near-infrared spectroscopy (FS/NIRS) and hyperspectral imaging (HSI) have emerged for the quick and easy detection of AFs (Tao et al., 2018).

Hussain et al. (2015) utilized the PCR technique for the molecular detection of AF producing *A. flavus* from peanuts. Similarly, the avfA, omtA, and ver-1 genes encoding the major enzymes in AF-biosynthesis were used as target genes for detecting AFs using multiplex PCR (Yang et al., 2004). Further, PCR was employed to detect AF-producing genes in *Aspergillus* species in Iranian pistachio nuts for their aflatoxigenic effect (Rahimi et al., 2008). In addition, Kim et al. (2014) utilized PCR, ELISA and HPLC for the detection of AFs from *A. oryzae* isolated from different Korean foods. HSI uses the integration of both imaging and spectroscopy to record spatial and spectral characteristics of a given sample (Wu and Sun, 2013; Ropodi et al., 2016; Shrestha et al., 2016; Siche et al., 2016). The visible/near-infrared (VNIR) HSI has been utilized for the identification of maize kernels of different varieties (Zhang et al., 2012; Wang et al., 2016). VNIR or short-wave (SWNIR) HSI techniques are feasible for the detection of AFs as well as identification of different fungal species in maize (Pearson and Wicklow, 2006; Williams et al., 2012; Wang et al., 2015a, b). Later, Kimuli et al. (2018b) used the VNIR-HSI system to detect AFB1 on surfaces of maize kernels from Georgia, Illinois, Indiana and Nebraska of United States. Chu et al. (2017) used short-wave infrared (SWIR) HIS to detect AFB1 in single maize kernels. But as the image quality could not effectively classify AFB1 level qualitatively in individual maize kernels, therefore, to improve this Kimuli et al. (2018a) further combined the SWIR-HSI system with chemometric data analysis for the better detection of AFB1 on the surfaces of maize kernels. Furthermore, the color-encoded lateral flow immunoassay (LFIA) technique has been used for the simultaneous detection of AFB1 as well as fumonisins in a single test line (Di Nardo et al., 2019).

To further enhance the sensitivity and detection of AFs in food and feed, nanoparticles (NPs) based on Au/Ag, carbon (CBNs), magnetic (MNPs), Quantum dots (QDs), up-conversion (UCNPs), metal-organic frameworks (MOFs) as well as hybrid nanostructures have been utilized (Xue et al., 2019). Rui et al. (2019) prepared molecular imprinted polymers (FDU-12@MIPs) using structural analog of AFs. This highly selective surface was used as an extraction sorbent in conjunction with HPLC for the detection of AFs in different food and feed samples. In addition to this, the use of biosensors compared to other spectrophotometric or chromatographic methods allow for higher selectivity, direct detection with minimal sample pretreatment, minimal cost, portability and on-field analysis of mycotoxins (Rotariu et al., 2016). Selvolini et al. (2019) utilized an electrochemical enzyme-linked oligonucleotide array for easy and quick multi-detection of AFB1 in maize. Furthermore, assays based on aptamer have been developed for the rapid detection of AFB1. Wang et al. (2019) successfully detected the AFB1 spiked in wine, methanol and corn flour samples using the simple aptamer molecular beacon assay, which has the potential for the rapid detection of AFs in the food and feed.

## Masked Mycotoxins as a Major Concern in Detection

Masked mycotoxins pose a major concern in food and feed as they are not identified and detected by the usually employed detection techniques (Kamle et al., 2019). These are the mycotoxins produced by fungi but are modified by plant enzymes during the infection stages. They are present in vacuoles in the soluble form or bound to macromolecules, therefore, are unable to be identified by routine analysis processes and referred to as masked mycotoxins (Berthiller et al., 2013). However, the modified AFs can hydrolyze back into the toxic forms during food processing and/or digestion process (Gareis et al., 1990; Nagl et al., 2014; Broekaert et al., 2015). Some of these modified toxins are present in different forms as complexes with matrix compounds, hence also referred to as matrix-associated mycotoxins (Rychlik et al., 2014). The masked mycotoxins have been reported to occur in Asia, Africa, America and Europe. Therefore, a high amount of masked mycotoxins prevailing in various food and feed can pose serious health issues to both humans and animals (Zhang et al., 2019). Therefore, the detection of masked mycotoxins is an essential part to ensure food and feed safety. Masked fumonisins were determined through hydrolysis where modified forms were converted back to their free forms and subsequently analyzed and detected through LC/MS/MS (Dall’Asta et al., 2008; Dall’Asta et al., 2009). The hydrolytic process may involve either alkaline, acidic or enzymatic treatments (Dall’Asta et al., 2009; Beloglazova et al., 2013; Vidal et al., 2018). However, there is less information available on the masked AFs as most of the preference is given for the detection of free AFs in agricultural food and feed. Therefore, methods like *in vitro* digestion and hydrolysis, as applied in case of masked fumonisins, can be carried out for masked AFs in food and feed followed by detection with LC/MS/MS and confirmation by other methods like ELISA to ensure the food and feed safety.

## Control Strategies of Aflatoxins

Implementation of advanced agricultural technologies, good agricultural practices (GAPs), good manufacturing practices (GMPs) and good storage practices (GSPs) can mitigate the mycotoxins contamination (Kamle et al., 2019). The novel processing techniques involving a microwave, UV, pulsed light, electrolyzed water, cold plasma, ozone, electron beam and gamma (γ) irradiation treatment have the potential for AFs management and preserving and maintaining the quality of agricultural and food products (Jalili et al., 2010; Pankaj et al., 2018). The application of ozone degrades AFs by an electrophilic attack on the double-bonded carbons (C8-C9) of the furan ring resulting in the formation of primary ozonides followed by rearrangement into monozonide derivatives like aldehydes, ketones and organic acids (Jalili, 2016). Further, the detailed mechanism of ozone degrading AFB1 has been discussed by Diao et al. (2013). The application of ozone for the degradation AF is limited in food products due to the cost factor (Womack et al., 2014). Similarly, the mechanism behind the AF degradation by gamma rays lies on the effects of free radicals produced during the radiolysis of water and other components that attacks the terminal furan ring of AFB1 resulting in byproducts of reduced biological activity (Rustom, 1997). The degradation efficiency of gamma irradiation is more effective when combined with other technologies.

In addition to these, several synthetic and natural food additives have been studied for AFs reduction in food and feed. For examples, the use of citric acid in combination with moisture under high temperature (200°C) and pressure (8N) was effective in degrading AFs in extruded sorghum (Méndez-Albores et al., 2009). On the other hand, the efficacy of sodium hydrosulphite (Na_2_S_2_O_4_) was enhanced with increased pressure for AFs reduction in black pepper (Jalili and Jinap, 2012). Furthermore, as a part of biological control measures, Anjaiah et al. (2006) reported that inoculation of antagonistic strains of *Pseudomonas, Bacillus* and *Trichoderma* spp. had a significant reduction of *A. flavus* in pre-harvest crops. The non-aflatoxin forming strains of *A. flavus* and other non-toxigenic molds are prominent biological control agents against AFs contamination (Dorner et al., 2003; Udomkun et al., 2017). The application of each technique has its advantages and disadvantages. Therefore, biocontrol measures in synchrony with other physical and chemical methods along with improved packaging materials should be implemented to attain food safety and security.

## Conclusion

Aflatoxins’ contamination of crops at pre- and post-harvest conditions can be controlled to some extent by the implementation of good agricultural practices (GAPs), good manufacturing practices (GMPs) and good storage practices (GSPs). Further, the novel processing technologies involving a microwave, UV, pulsed light, electrolyzed water, cold plasma, ozone, electron beam or gamma (γ) irradiation in combination with either biological, physical, chemical or genetic engineering methods have the potential to improve the efficiency of AFs decontamination as well as to overcome the limitations of any specific technology. However, it is vital to understand the mechanisms of AFs detoxification so that no AF-residues are left behind when these methods are applied in food and feed samples. Furthermore, as there is less information on the masked AFs present in food and feed, it requires in-depth research and understanding with regards to adequate hydrolysis, identification, detection and control strategies. Therefore, utilization of the novel technologies along with raising public awareness for implementing GAPs, GMPs and GSPs are crucial for controlling AFs contamination in food and feed to ensure food safety and security and to safeguard human and animal health.

## Author Contributions

PK conceived and designed the manuscript. DM, PK, and MK wrote the manuscript. KL, KD, and SD helped in the editing of the manuscript. PK and SK critically reviewed the manuscript and did the required editing.

## Conflict of Interest

SD employed by company SAB Miller India Ltd., Sonipat, India. The remaining authors declare that the research was conducted in the absence of any commercial or financial relationships that could be construed as a potential conflict of interest.
